# Nicotinamide mononucleotide supplementation improves the quality of porcine oocytes under heat stress

**DOI:** 10.1186/s40104-022-00716-0

**Published:** 2022-06-15

**Authors:** Meijie Song, Yu Li, Yihang Zhou, Jiner Yan, Xiaohua Zhou, Qian Gao, Yilong Miao, Bo Xiong

**Affiliations:** 1 College of Animal Science and Technology, Nanjing Agricultural University, Nanjing, 210095 China; 2 College of Veterinary Medicine, Nanjing Agricultural University, Nanjing, 210095 China

**Keywords:** Heat stress, Meiotic defects, Nicotinamide mononucleotide, Oocyte quality, Porcine oocytes

## Abstract

**Background:**

Elevated ambient temperature-caused heat stress is a major concern for livestock production due to its negative impact on animal feed intake, growth, reproduction, and health. Particularly, the germ cells are extremely sensitive to the heat stress. However, the effective approach and strategy regarding how to protect mammalian oocytes from heat stress-induced defects have not been determined.

**Methods:**

Germinal vesicle (GV) porcine oocytes were cultured at 41.5 °C for 24 h to induce heat stress, and then cultured at 38.5 °C to the specific developmental stage for subsequent analysis. Nicotinamide mononucleotide (NMN) was dissolved in water to 1 mol/L for a stock solution and further diluted with the maturation medium to the final concentrations of 10 μmol/L, 20 μmol/L, 50 μmol/L or 100 μmol/L, respectively, during heat stress. Immunostaining and fluorescence intensity quantification were applied to assess the effects of heat stress and NMN supplementation on the key processes during the oocyte meiotic maturation.

**Results:**

Here, we report that NMN supplementation improves the quality of porcine oocytes under heat stress. Specifically, we found that heat stress resulted in oocyte maturation failure by disturbing the dynamics of meiotic organelles, including the cytoskeleton assembly, cortical granule distribution and mitochondrial function. In addition, heat stress induced the production of excessive reactive oxygen species (ROS) and DNA damage, leading to the occurrence of apoptosis in oocytes and subsequent embryonic development arrest. More importantly, we validated that supplementation of NMN during heat stress restored the meiotic defects during porcine oocyte maturation.

**Conclusions:**

Taken together, our study documents that NMN supplementation is an effective approach to improve the quality of oocytes under heat stress by promoting both nuclear and cytoplasmic maturation.

## Introduction

Constant temperature is critical for the normal physiological functions of organisms [1]. However, animals usually suffer from heat stress during hot weather or environment, particularly when temperatures are extremely high and the exposure time is long. Heat stress negatively affects human health, animal growth, and livestock production [2]. It has been reported that heat stress causes the mitochondrial damage in rat cardiomyocytes, thereby leading to the cardiac dysfunction and failure [3]. Chronic heat stress induces the expression of liver proteins involved in immune response, oxidative stress response and apoptosis, which compromises the growth of porcine liver [4]. Additionally, it is worth noting that the reproductive system of animals appears more sensitive to heat. The testicular temperature in most male animals is lower than their body temperature. Thus, excessive heat is prone to impair spermatogenesis and may cause the male infertility [5]. It has also been shown that the growing ovine oocytes are arrested in the germinal vesicle breakdown (GVBD) stage with an aberrant chromatin configuration under heat stress [6]. The porcine and bovine oocytes exposed to heat stress fail to be matured in vitro due to the defects in the cytoskeleton organization, and obtain the reduced potential for early embryo development [7–10]. Importantly, heat stress adversely affects the mitochondrial function to induce the production of reactive oxygen species (ROS) and oxidative stress, lower the antioxidant capacity, and result in DNA damage and apoptosis in the cells [11, 12].

Nicotinamide adenine dinucleotide (NAD^+^) is a classical coenzyme that plays a critical role in energy metabolism to maintain the proper cell functioning and participate in various biological processes, such as metabolism, inflammation, circadian rhythm, and aging [13, 14]. It has been demonstrated that the levels of NAD^+^ decrease with age, leading to the mitochondrial dysfunction and abnormal metabolism in the organism [15–18]. Nicotinamide mononucleotide (NMN) is a naturally occurring nucleotide precursor of NAD^+^, which is produced through the NAD^+^ rescue pathway [19]. Recovery of NAD^+^ levels by supplementing NMN can substantially ameliorate the age-related functional defects, and thus counteracting the aging diseases [14, 20, 21]. A previous study has shown that NMN restores the function of cerebrovascular endothelium to improve cognitive function in aged mice [22]. NMN also reverses vascular dysfunction by activating SIRT1 activity and decreasing oxidative stress in aged mice [21]. Furthermore, we recently reported that NMN supplementation rejuvenates the quality of maternally aged mouse oocytes and rescues the female fertility [23]. However, whether NMN could improve the quality of oocytes under environmental stress has not been determined.

In the present study, we used porcine oocytes as a research model to study the effect of NMN on the quality of oocytes under heat stress. We found that heat stress caused the porcine oocyte meiotic failure by perturbing both nuclear and cytoplasmic maturation. NMN supplementation effectively inhibited the increase in the occurrence of DNA damage and apoptosis induced by heat stress and restored the quality of porcine oocytes.

## Materials and methods

### Porcine oocyte collection and in vitro maturation

Abattoir-derived porcine ovaries were transported to the laboratory within 2 h in a physiological saline containing penicillin G/streptomycin sulphate. Cumulus-oocyte complexes (COCs) were isolated from the follicles (3–6 mm in diameter) using a disposable syringe with a 20-gauge needle. Oocytes with compact cumulus cells were used for in vitro maturation (IVM) in TCM-199 (ThermoFisher Scientific, Waltham, MA, USA) supplemented with 10 ng/mL EGF, 5 μg/mL insulin, 0.2 mmol/L pyruvate, 0.6 mmol/L cysteine, 10% porcine follicular fluid, 10 IU/mL of each eCG and hCG, and 25 μg/mL kanamycin. 20–30 germinal vesicle (GV) oocytes were cultured for 26–28 h to metaphase I stage and for 44–48 h to metaphase II stage in 100 μL TCM-199 covered with mineral oil at 38.5 °C, 5% CO2.

### Heat treatment and NMN supplementation

The procedure for heat treatment referenced to a previous study [24]. In short, GV oocytes were cultured at 41.5 °C for 24 h for heat stress, and then cultured at 38.5 °C to the specific developmental stage for subsequent analysis. NMN (GeneHarbor Biotech, Hong Kong, China) was dissolved in water to 1 mol/L for a stock solution and further diluted with the maturation medium to the final concentrations of 10 μmol/L, 20 μmol/L, 50 μmol/L or 100 μmol/L, respectively, during heat stress.

### Fluorescence staining and imaging

For immunostaining, denuded oocytes were fixed in 4% paraformaldehyde/PBS (fixation solution) for 30 min and then transferred to 1% Triton X-100/PBS (permeabilization solution) for 8 h. After washing in PBST, oocytes were incubated with 3% BSA/PBS (blocking solution) for 1 h at room temperature (RT), followed by incubation with α-tubulin-FITC antibody (1:200; Sigma-Aldrich, St. Louis, MO, USA), acetyl-α-tubulin antibody (1:100; Sigma-Aldrich), human ovastacin antibody (1:100; Jurrien Dean lab, NIH), γH2A.X antibody (1:100; Cell Signaling Technology, Danvers, MA, USA), phalloidin-TRITC (1:100; Sigma-Aldrich) and LCA-FITC (1:100; Sigma-Aldrich) at 4 °C overnight. After three times of washes in PBST, oocytes were incubated with corresponding secondary antibodies for 1 h and counterstained with 10 μg/mL propidium iodide (PI) or Hoechst 33342 for 10 min at RT. For dye staining, oocytes were stained with 500 nmol/L MitoTracker Red CMXRos (ThermoFisher Scientific) for 30 min at 38.5 °C for mitochondrial distribution, with 2 μmol/L MitoProbe JC-1 (ThermoFisher Scientific) for mitochondrial membrane potential evaluation, with 10 μmol/L dichlorofluorescein diacetate (DCFH-DA) for ROS level, and with Annexin-V-FITC (1:10; Beyotime, Huangzhou, China) for apoptosis observation. Finally, oocytes were mounted on the glass slides and imaged by the laser confocal microscope (LSM 900 META, Zeiss, Germany).

### RNA sequencing

Metaphase II oocytes after NMN treatment were collected from control, heat-stressed and NMN-supplemented groups (100 oocytes per group), and total RNA was extracted using RNeasy Micro Kit (Qiagen) according to manufacturer’s instructions. Extracted RNA was quantified with the Qubit RNA Assay Kit (ThermoFisher Scientific). mRNA library construction was performed with NEBNext Ultra RNA Library Prep Kit for Illumina (New England Biolabs) according to the manuals. The protocol consisted of sequential RNA fragmentation, reverse transcription using random primers, second strand cDNA synthesis, end repair, dA-tailing, adapter ligation, and PCR enrichment. The concentration and quality of libraries were tested by a NanoDrop 2000 spectrophotometer (ThermoFisher Scientific), qPCR, and Agilent 2100 Bioanalyzer (Agilent Technologies, Palo Alto, CA, USA). Then the libraries were sequenced on Illumina Hiseq X Ten instruments with 150 bp pair-end reads. High-quality sequences (clean reads) were obtained by trimming adaptor sequences and removing low-quality reads from raw RNA-seq reads using cutadapt (v1.10). Reads were then aligned to the susScr11 reference genome using tophat2 (v2.0.13).

### RNA isolation and quantitative real-time PCR

Total RNA was extracted from 30 oocytes using RNeasy Mini Kit (Qiagen, Germantown, MD, USA) and reversed to cDNA using PrimeScript RT Master Mix (Takara, Kusatsu, Shiga, Japan), followed by storing at − 20 °C until use. Quantitative real-time PCR was conducted using SYBR Green PCR Master Mix with QuantStudio 7 Flex Real-Time PCR System (ThermoFisher, Waltham, MA, USA). Data were normalized against *GAPDH* and quantification of the fold change was determined by the comparative CT method. The primers were listed as follows:

*CSNK2B* (F: 5'-GTCCAGCCGCTGAAGTGAA-3'/R: 5'-GCTCGTTGAGTCCAGTGAGA-3');

*MED25* (F: 5'-TCCTGTACTCGTCCAAGAAGAAG-3'/R: 5'-CTGCTGGACTTGCTTGTGGTT-3');

*KDM6NB* (F: 5'-TTCGCGTCCTACATGACCAC-3'/R: 5'-TCAGCGTCAGCTTGATGTGT-3');

*PLK3* (F: 5'-AAAACAGTCCGCAGGTCCAT-3'/R: 5'-TGGTCGGGTCTCATCAATGC-3');

*GAPDH* (F: 5'-AGGTCGGTGTGAACGGATTTG-3'/R: 5'-TGTAGACCATGTAGTTGAGGTCA-3').

### Parthenogenetic activation and early embryonic development

After 44 h of in vitro culture, metaphase II oocytes were washed in the activation medium (0.3 mol/L Mannitol, 0.5 mmol/L HEPES, 0.1 mmol/L MgSO_4_•7H_2_O, 0.05 mmol/L CaCl_2_) at 38.5 °C. Then oocytes were electrically activated in a microslide with 0.5 mm fusion chamber using a single direct current pulse of an electrical pulse (0.9 KV/cm, 80 μs, CRY-3B Cell Fusion Instrument, Ningbo, China), followed by the chemical activation with cytochalasin B (5 mg/mL) and cycloheximide (1 mg/mL) in PZM-3 medium for 4 h. The activated oocytes were further cultured in PZM-3 medium at 38.5 °C, 5% CO2 for 6 d to observe the blastocyst formation.

### Statistical analysis

All percentages or values from at least three independent replicates were expressed as mean ± SEM or mean ± SD, and the number of oocytes observed was labeled in parentheses as (*n*). Data were analyzed by paired-samples *t*-test, provided by GraphPad Prism 8 statistical software. The level of significance was accepted as *P* < 0.05.

## Results

### NMN recovers the meiotic failure of porcine oocytes under heat stress

**Fig. 1:**
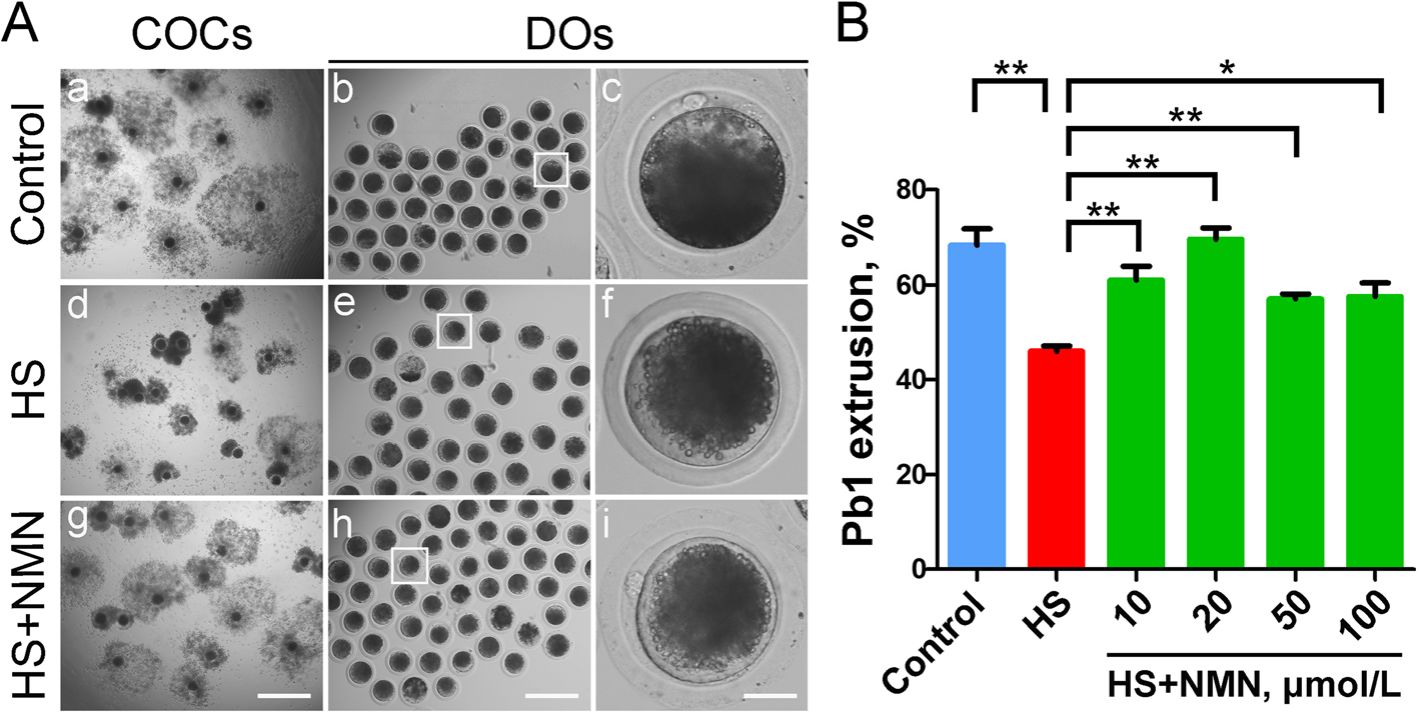
Effects of NMN supplementation on the maturation of heat-stressed porcine oocytes. **A** In vitro maturation of oocytes in control, heat-stressed (HS) and NMN-supplemented (HS + NMN) groups. Cumulus cell expansion of cumulus-oocyte complexes (COCs) and polar body extrusion (PBE) of denuded oocytes (DOs) were imaged by the confocal microscope. Scale bar, 400 μm (a, d, g); 250 μm (b, e, h); 30 μm (c, f, i). **B** The rates of PBE were recorded in control, heat-stressed and NMN-supplemented (10 μmol/L, 20 μmol/L, 50 μmol/L and 100 μmol/L) oocytes after culture for 44 h in vitro. Data were presented as mean percentage (mean ± SEM) of at least three independent experiments. *P < 0.05, **P < 0.01

To determine the effect of heat stress on the meiotic progression of porcine oocytes, we exposed GV oocytes at 41.5 °C for 24 h, and then cultured them at 38.5 °C for another 20 h. After 44 h of in vitro maturation, we assessed the expansion of cumulus cells surrounding cumulus-oocyte complexes (COCs) and the rate of polar body extrusion (PBE). As shown in Fig. 1A, in the control group, the cumulus cells of most COCs were completely expanded, with a large and uniform expansion area. While in the heat-stressed group, the expansion ability of cumulus cells was significantly weakened, by displaying partially or entirely no expansion (Fig. 1A). Quantitative data showed that the proportion of PBE in heat-stressed group was considerably decreased compared to the control group (68.3 ± 3.5%, *n* = 59 vs. 46.0 ± 1.1%, *n* = 74, *P* < 0.01; Fig. 1B).

We next asked whether supplementation of NMN could improve the heat stress-induced oocyte maturational defect. For this purpose, different concentrations of NMN (10, 20, 50 and 100 μmol/L) were added to the culture medium for in vitro maturation of oocytes under heat stress. The results revealed that supplementation with 20 μmol/L NMN has the best rescue effect, not only improving the expansion of cumulus cells surrounding COCs, but also considerably increasing the proportion of PBE in heat-stressed oocytes (46.0 ± 1.1%, *n* = 74 vs. 69.5 ± 2.4%, *n* = 81, *P* < 0.01; Fig. 1A, B). Taken together, these observations indicate that NMN can, at least partially, restore the failure of porcine oocyte meiotic progression caused by heat stress.

### NMN maintains the spindle/chromosome structure in porcine oocytes under heat stress

**Fig. 2:**
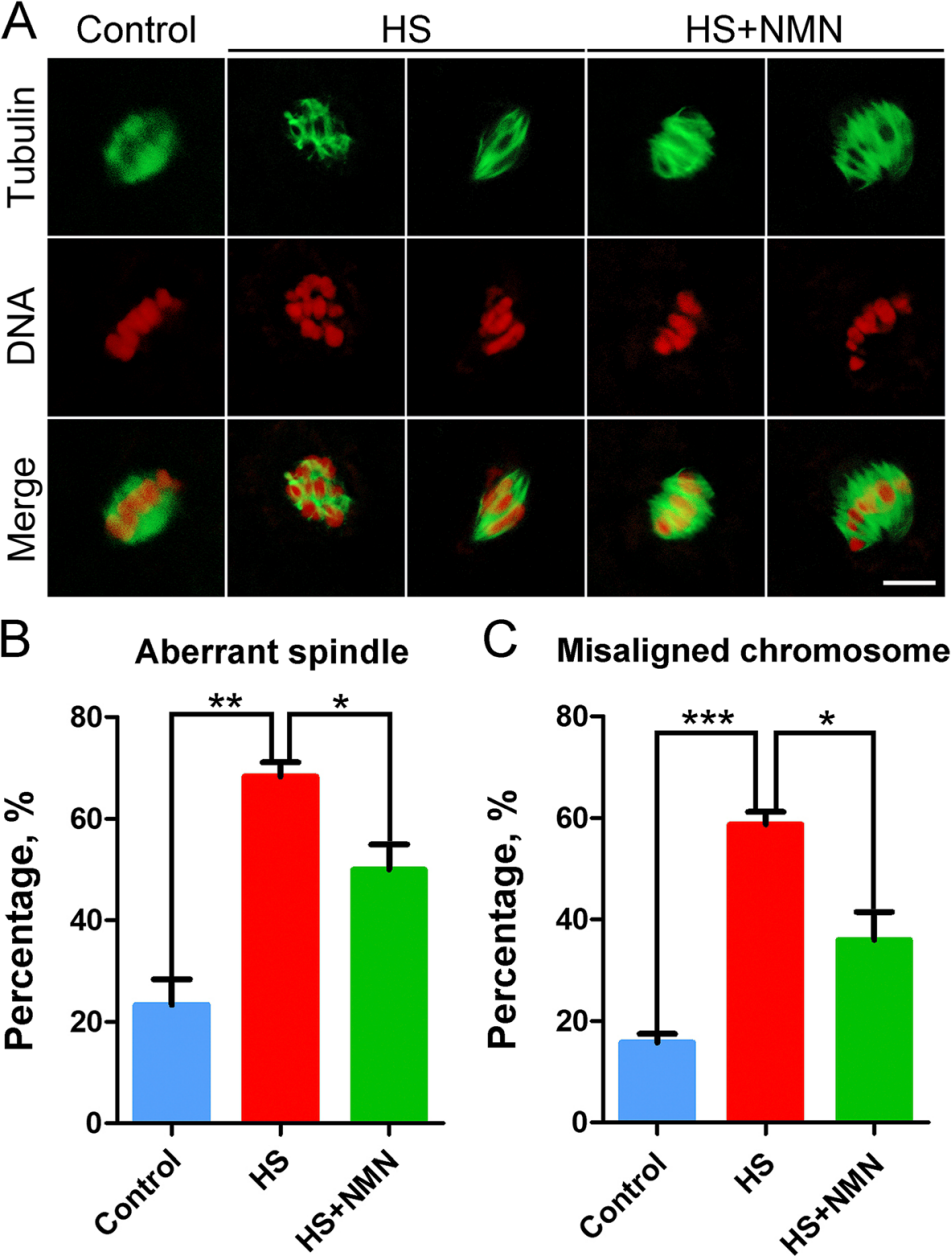
Effects of NMN supplementation on the spindle assembly and chromosome alignment in heat-stressed porcine oocytes. **A** Representative images of spindle/chromosome structure in control, heat-stressed and NMN-supplemented oocytes at metaphase I stage. Scale bar, 5 μm. **B** The rate of disorganized spindles was recorded in control, heat-stressed and NMN-supplemented oocytes. **C** The rate of misaligned chromosomes was recorded in control, heat-stressed and NMN-supplemented oocytes. Data in (**B**) and (**C**) were presented as mean percentage (mean ± SEM) of at least three independent experiments. *P < 0.05, **P < 0.01, ***P < 0.001

To explore whether the failure of porcine oocyte meiosis under heat stress is caused by spindle/chromosome abnormalities, metaphase I (M I) oocytes were stained with anti-α-tubulin-FITC antibody to show the spindle apparatus and counterstained with propidium iodide (PI) to display the chromosome alignment. A standard barrel-shaped spindle structure with well-aligned chromosomes at the equatorial plate was present in control oocytes (Fig. 2A). However, the frequency of disorganized spindles such as non-polar, multi-polar, elongated or dispersive ones with misaligned chromosomes was prominently higher in heat-stressed oocytes than that in controls (spindle: 23.3 ± 5.1%, *n* = 31 vs. 68.4 ± 2.8%, *n* = 43, *P* < 0.01; chromosome: 15.8 ± 1.7%, *n* = 31 vs. 58.8 ± 2.5%, *n* = 43, *P* < 0.001; Fig. 2B, C). When supplemented with NMN in the culture medium, the abnormal rate of spindle morphology and chromosome alignment in heat-stressed oocytes was significantly reduced (spindle: 50.0 ± 5.0%, *n* = 30, *P* < 0.05; chromosome: 46.0 ± 5.5%, *n* = 30, *P* < 0.05; Fig. 2A-C), suggesting that NMN supplementation promotes the maturation of porcine oocytes under heat stress by maintaining the spindle/chromosome structure.

### NMN stabilizes microtubules in porcine oocytes under heat stress

**Fig. 3:**
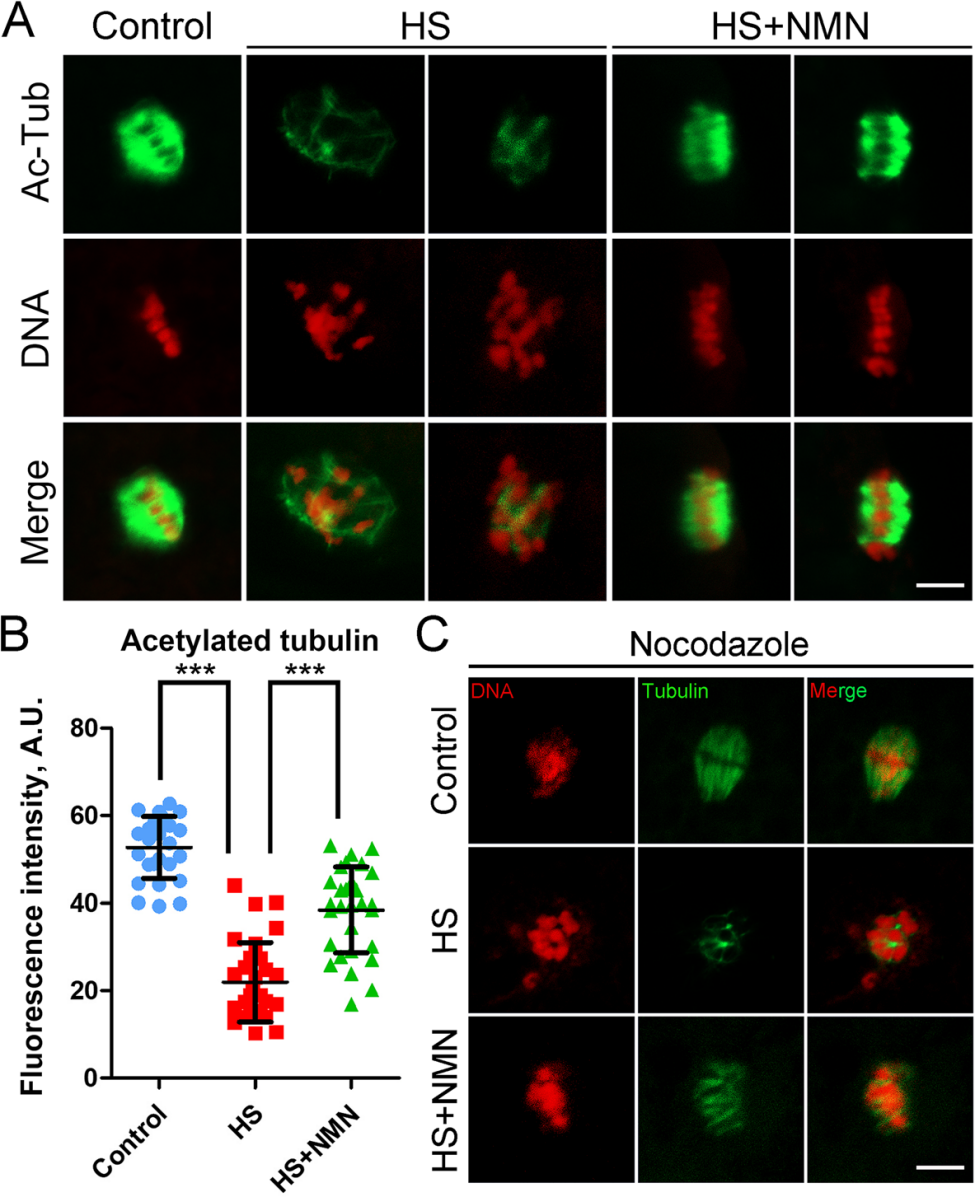
Effects of NMN supplementation on the microtubule stability in heat-stressed porcine oocytes. **A** Representative images of acetylated α-tubulin in control, heat-stressed and NMN-supplemented oocytes at metaphase I stage. Scale bar, 5 μm. **B** The fluorescence intensity of acetylated α-tubulin was quantified in control, heat-stressed and NMN-supplemented oocytes. Data were presented as mean value (mean ± SD) of at least three independent experiments. ***P < 0.001. **C** Representative images of microtubule fibers after nocodazole treatment in control, heat-stressed and NMN-supplemented oocytes. Oocytes were placed in cold culture media at 4 °C for 5 min, followed by the immunostaining for α-tubulin. Scale bar, 5 μm

As microtubule dynamics is responsible for the regulation of spindle assembly, we next explored if the defects of spindle assembly in heat-stressed oocytes are caused by the impaired microtubule stability. We then evaluated the acetylation level of α-tubulin, an important indicator for the stability of microtubules. As judged by the immunofluorescence analysis, we observed that heat stress greatly reduced the signals of acetylated α-tubulin compared to the controls (52.7 ± 1.4, *n* = 27 vs. 22.0 ± 1.7, *n* = 30, *P* < 0.001; Fig. 3A, B). On the contrary, NMN supplementation significantly increased the acetylation level of α-tubulin in heat-stressed oocytes (38.4 ± 1.8, *n* = 30, *P* < 0.001; Fig. 3A, B), suggesting that NMN could restore the disturbed spindle assembly in porcine oocytes under heat stress by maintaining microtubule stability.

To further verify the defective microtubule stability in heat-stressed oocytes, we used the microtubule depolymerization drug nocodazole to collapse the microtubules. As shown in Fig. 3C, in the control oocytes, after treatment with 20 μg/mL nocodazole for 5 min, although the spindle morphology was destroyed, the microtubule fibers still existed. However, by performing the same nocodazole treatment, the microtubule fibers completely disappeared in heat-stressed oocytes, indicative of the attenuated microtubule stability. As anticipated, NMN supplementation prominently strengthened the stability of microtubule fibers under heat stress (Fig. 3C).

### NMN protects the dynamic polymerization of actin cytoskeleton from damage in porcine oocytes under heat stress

**Fig. 4:**
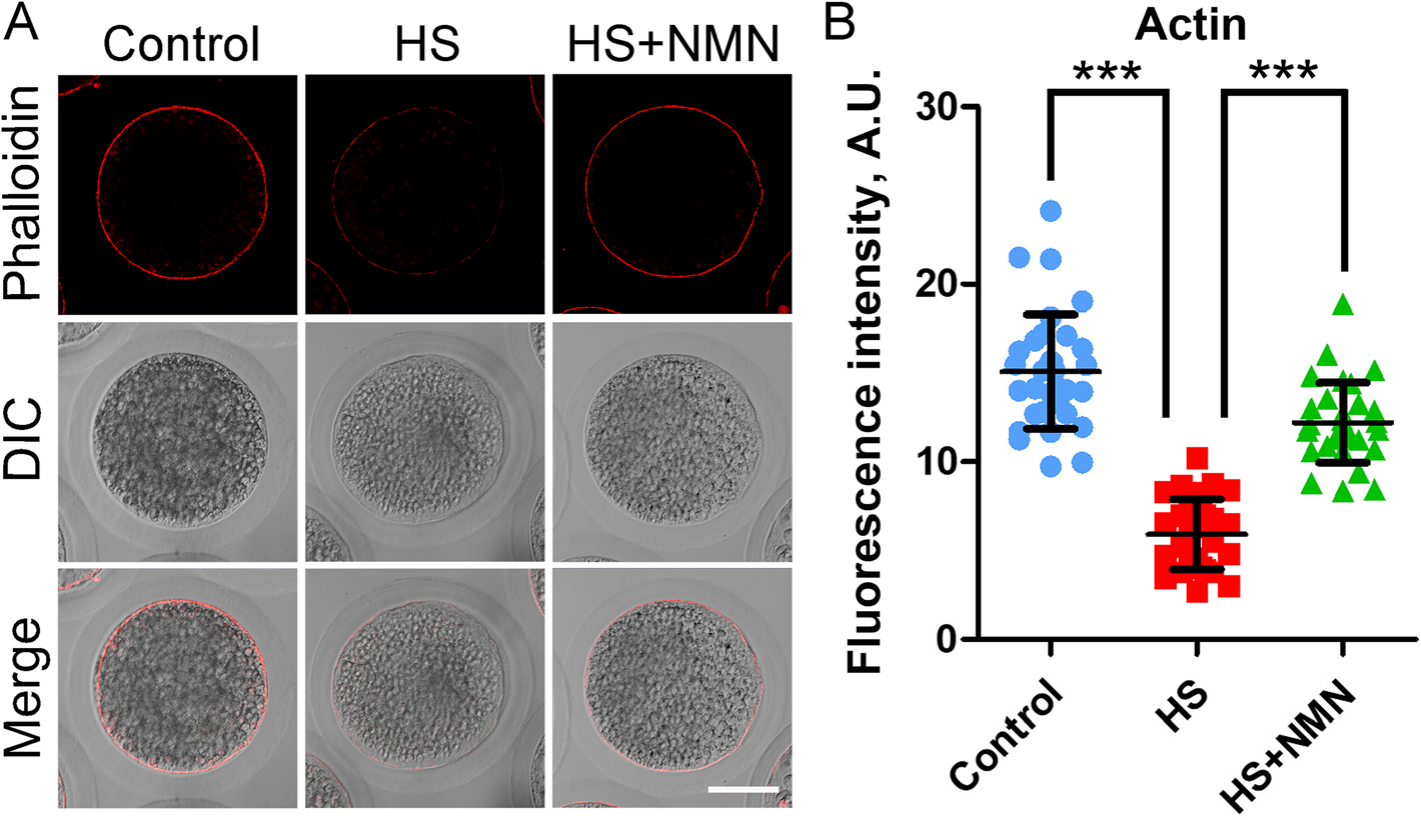
Effects of NMN supplementation on the actin polymerization in heat-stressed porcine oocytes. **A** Representative images of actin filaments in control, heat-stressed and NMN-supplemented oocytes at metaphase I stage. Scale bar, 30 μm. **B** The fluorescence intensity of actin signals was quantified in control, heat-stressed and NMN-supplemented oocytes. Data were presented as mean value (mean ± SD) of at least three independent experiments. ***P < 0.001

In oocyte meiosis, actin filaments play a critical role in the maintenance of cell surface structure and spindle migration [25]. To determine whether the failure of porcine oocyte maturation caused by heat stress is linked with actin filaments, F-actin was stained with phalloidin-TRITC. In control oocytes, actin was uniformly distributed on the plasma membrane and showed strong fluorescence signals (Fig. 4A). In sharp contrast, in heat-stressed oocytes, the fluorescence signals of actin were weak and presented a discontinuous distribution (Fig. 4A). Whereas, supplementation with NMN restored the actin dynamics disrupted by heat stress (Fig. 4A). In addition, the quantitative data of fluorescence signal intensity confirmed above observations (15.1 ± 0.6, *n* = 33, *P* < 0.001 vs. 5.9 ± 0.4, *n* = 30 vs. 12.2 ± 0.4, *n* = 31, *P* < 0.001; Fig. 4B), indicating that NMN recovers the actin dynamics in porcine oocytes under heat stress.

### NMN rescues the distribution of CGs and ovastacin in porcine oocytes under heat stress

**Fig. 5:**
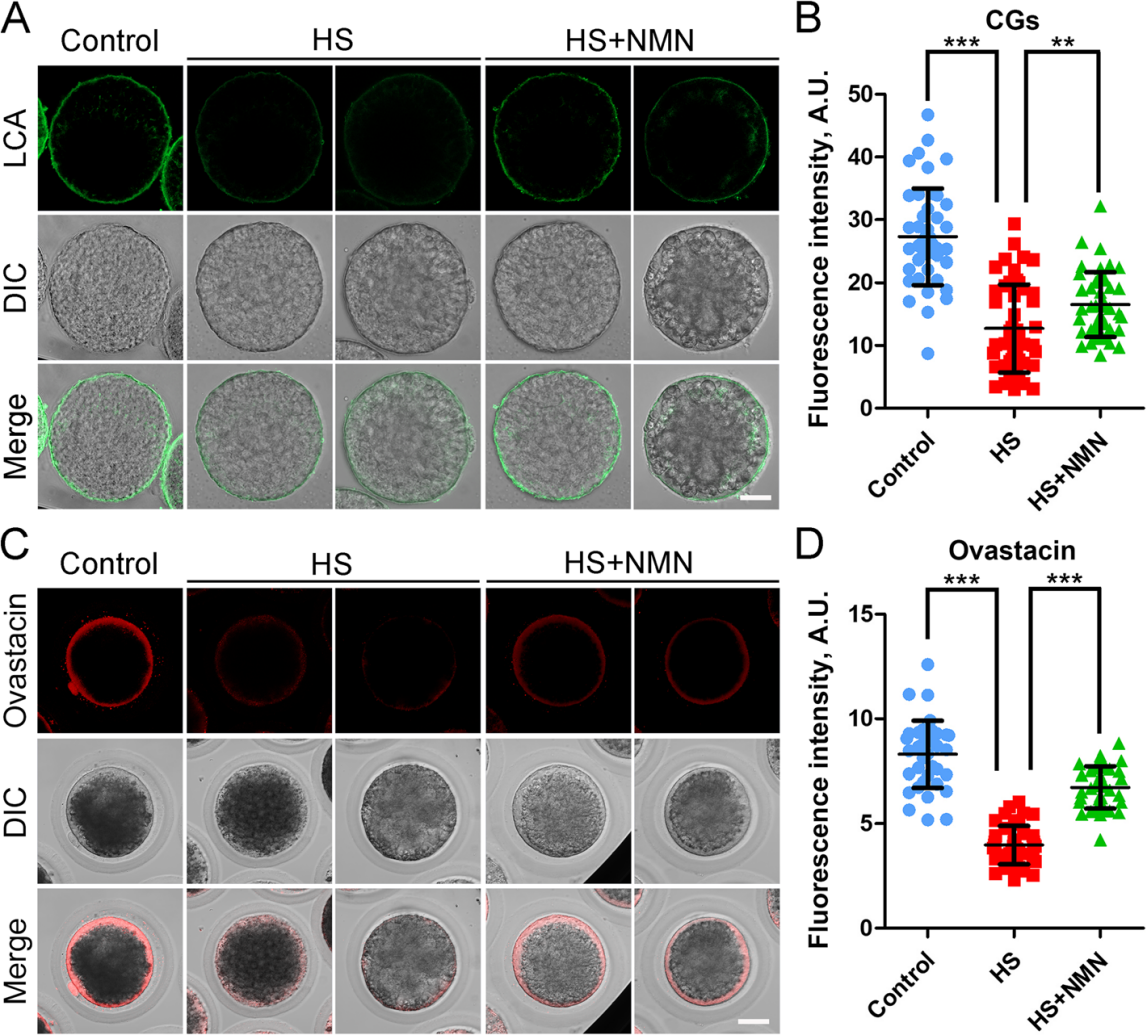
Effects of NMN supplementation on the distribution of CGs and ovastacin in heat-stressed porcine oocytes. **A** Representative images of CG localization in control, heat-stressed and NMN-supplemented oocytes at metaphase II stage. Zona pellucida was removed from oocytes before LCA-FITC staining because of the non-specific binding. Scale bar, 30 μm. **B** The fluorescence intensity of CG signals was quantified in control, heat-stressed and NMN-supplemented oocytes. **C** Representative images of ovastacin localization in control, heat-stressed and NMN-supplemented oocytes at metaphase II stage. Scale bar, 30 μm. **D** The fluorescence intensity of ovastacin signals was quantified in control, heat-stressed and NMN-supplemented oocytes. Data in (B) and (D) were presented as mean value (mean ± SD) of at least three independent experiments. **P < 0.01, ***P < 0.001

Cortical granules (CGs) are a unique vesicular organelle in oocytes that function in the prevention of polyspermy [26], and their distribution under the subcortical region of oocytes has been considered as one of the key indicators of oocyte cytoplasmic maturation. The results in Fig. 5A showed that signals of CGs were remarkably weakened in oocytes after heat stress, but were partially restored by NMN supplementation. Quantitative analysis also revealed that the fluorescence intensity of CG signals in heat-stressed oocytes was significantly reduced in comparison with the controls (27.3 ± 1.1, *n* = 45 vs. 12.7 ± 1.0, *n* = 49, *P* < 0.001; Fig. 5B), and supplementation of NMN during in vitro culture increased the intensity (16.5 ± 0.8, *n* = 44, *P* < 0.01; Fig. 5B).

In agreement with above observations, we found that ovastacin, a component of CGs, exhibited a similar distribution pattern to CGs in control, heat-stressed and NMN-supplemented oocytes (Fig. 5C). Moreover, measurement of fluorescence intensity of ovastacin signals confirmed that ovastacin signals in the heat-stressed oocytes considerably decreased compared to the controls, but elevated after NMN supplementation (8.3 ± 0.3, *n* = 38, *P* < 0.001 vs. 4.0 ± 0.1, *n* = 43 vs. 6.7 ± 0.2, *n* = 40, *P* < 0.001; Fig. 5D). Altogether, these data suggest that NMN improves the cytoplasmic maturation of porcine oocytes under heat stress.

### Identification of target effector in NMN-supplemented oocytes under heat stress by transcriptome analysis

**Fig. 6:**
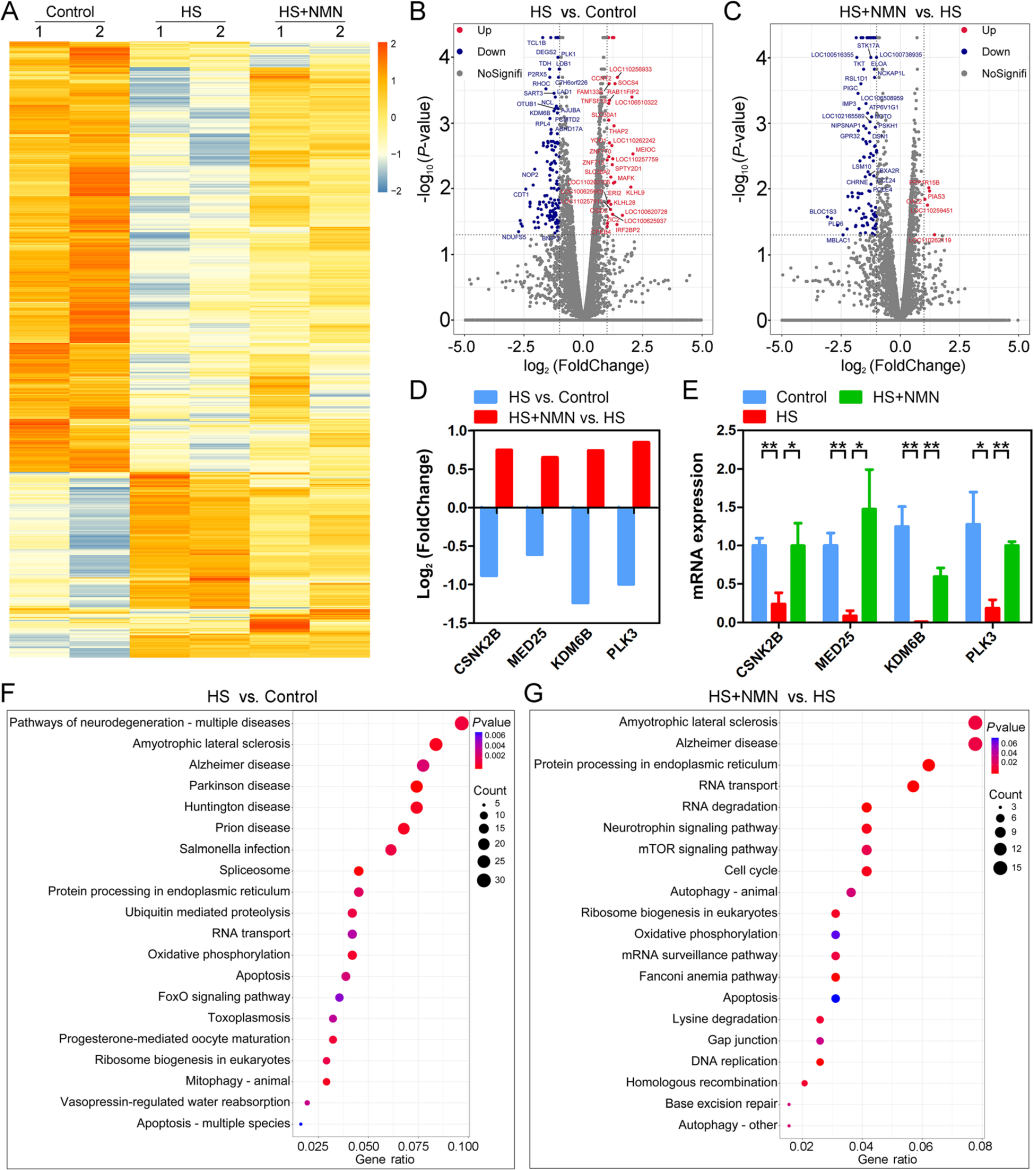
Effects of NMN supplementation on the transcriptome profiling of heat-stressed porcine oocytes. **A** Heatmap illustration showed the differentially expressed genes (DEGs) in control, heat-stressed and NMN-supplemented oocytes. **B** Volcano plot displayed the DEGs in heat-stressed oocytes compared to control oocytes. Red, upregulated; Blue, downregulated; Grey, not significant. **C** Volcano plot displayed the DEGs in NMN-supplemented oocytes compared to heat-stressed oocytes. Red, upregulated; Blue, downregulated; Grey, not significant. **D** RNA-seq results of selected genes in heat-stressed oocytes compared to control oocytes and NMN-supplemented oocytes compared to heat-stressed oocytes. **E** Validation of RNA-seq data by quantitative RT-PCR in control (blue), heat-stressed (red) and NMN-supplemented (green) oocytes. Data were presented as mean value (mean ± SEM) of at least three independent experiments. *P < 0.05, **P < 0.01. **F** KEGG pathway enrichment analysis of DEGs in heat-stressed oocytes compared to control oocytes. **G** KEGG pathway enrichment analysis of DEGs in NMN-supplemented oocytes compared to heat-stressed oocytes

To further understand the underlying mechanism regarding how NMN supplementation improves the quality of porcine oocytes under heat stress, we analyzed the transcriptome profiles of control, heat-stressed and NMN-supplemented oocytes by RNA sequencing (RNA-seq). The heat map and volcano plot data showed that a total of 953 differentially expressed genes (DEGs) were identified in heat-stressed oocytes compared to controls, and 537 DEGs were identified in NMN-supplemented oocytes compared to heat-stressed ones (Fig. 6A-C). The RNA-seq data were further verified by RT-PCR with four randomly selected genes (Fig. 6D, E). In particular, the KEGG pathway enrichment analysis revealed that DEGs in the oxidative phosphorylation and apoptosis pathways were enriched in both heat-stressed oocytes compared to control oocytes and NMN-supplemented oocytes compared to heat-stressed oocytes, suggesting that NMN might strengthen the mitochondrial function of heat-stressed oocytes to prevent the occurrence of apoptosis (Fig. 6F, G).

### NMN enhances the mitochondrial function in porcine oocytes under heat stress

**Fig. 7:**
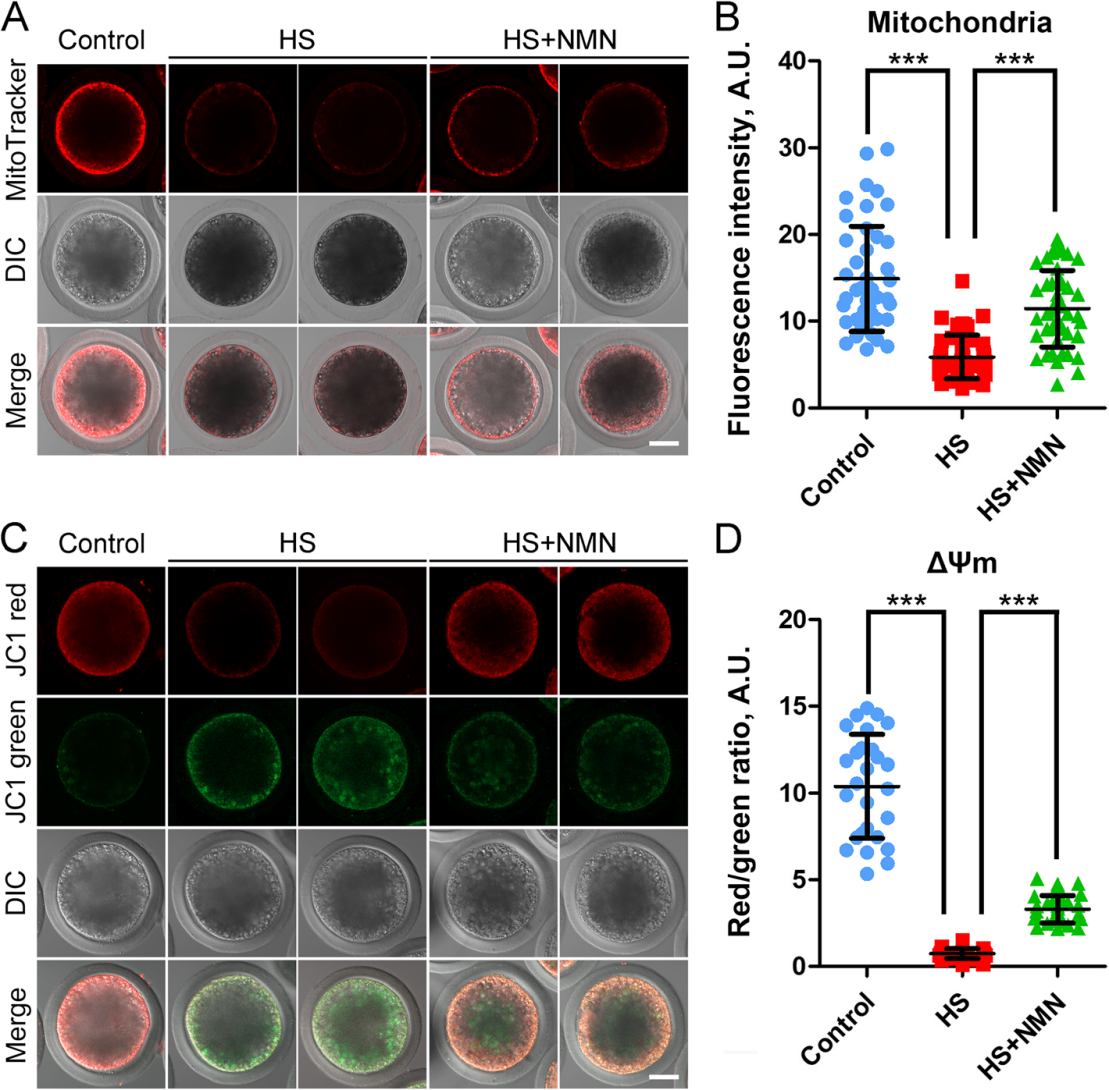
Effects of NMN supplementation on the mitochondrial distribution and function in heat-stressed porcine oocytes. **A** Representative images of mitochondrial distribution in control, heat-stressed and NMN-supplemented oocytes at metaphase II stage. Scale bar, 30 μm. **B** The fluorescence intensity of mitochondrial signals was quantified in control, heat-stressed and NMN-supplemented oocytes. **C** Mitochondrial membrane potential (ΔΨm) was detected by JC-1 staining in control, heat-stressed and NMN-supplemented oocytes at metaphase II stage. Red, high ΔΨm; Green, low ΔΨm. Scale bar, 30 μm. **D** The ratio of red to green fluorescence intensity was calculated in control, heat-stressed and NMN-supplemented oocytes. Data in (**B**) and (**D**) were presented as mean value (mean ± SD) of at least three independent experiments. ***P < 0.001

As transcriptome analysis has shown that mitochondrion might be the target organelle in which NMN functions, we next investigated the dynamic distribution of mitochondria by MitoTracker staining as previously described [27]. We observed that most of the mitochondria gathered in the subcortex area in control porcine oocytes, but reduced after heat stress (Fig. 7A). The correct positioning of mitochondria was partially restored in NMN-supplemented oocytes (Fig. 7A). By quantification of fluorescence intensity, the data indicated that mitochondrial signals in heat-stressed oocytes significantly decreased compared to the controls, but elevated after NMN supplementation (14.7 ± 0.9, *n* = 35, *P* < 0.001 vs. 2.3 ± 0.3, *n* = 31 vs. 6.4 ± 0.3, *n* = 34, *P* < 0.001; Fig. 7B).

We also evaluated the mitochondrial membrane potential (ΔΨm), an important indicator for mitochondrial function, by JC-1 staining [28]. Mitochondria with high ΔΨm showed red fluorescence, and with low ΔΨm showed green fluorescence (Fig. 7C). The quantitative analysis of the red to green fluorescence ratio displayed that of ΔΨm was much lower in heat-stressed oocytes than that in controls, but increased in NMN-supplemented oocytes (10.4 ± 0.6, *n* = 27, *P* < 0.001 vs. 0.7 ± 0.1, *n* = 32 vs. 3.3 ± 0.1, *n* = 36, *P* < 0.001; Fig. 7D). Taken together, these observations indicate that NMN boosts the mitochondrial dynamics and function in oocytes under heat stress.

### NMN suppresses the production of excessive ROS, DNA damage and apoptosis in porcine oocytes under heat stress

**Fig. 8:**
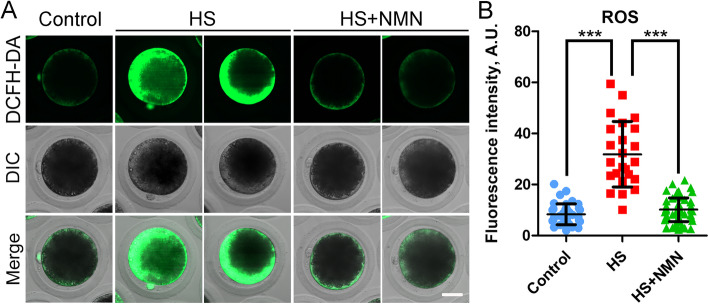
Effects of NMN supplementation on the ROS levels in heat-stressed porcine oocytes. **A** Representative images of ROS levels in control, heat-stressed and NMN-supplemented oocytes at metaphase II stage. Scale bar, 30 μm. **B** The fluorescence intensity of DCFH-DA signals were quantified in control, heat-stressed and NMN-supplemented oocytes. Data were presented as mean value (mean ± SD) of at least three independent experiments. ***P < 0.001

It has been reported that heat exposure induces oxidative stress in various cells, thereby impairing the normal physiological functions of cells and causing apoptosis [29, 30]. Combination with the transcriptome data, we speculated that the defects present in heat-stressed oocytes might be mediated by this mechanism as well. To verify this hypothesis, we compared the ROS levels in each group by dichlorofluorescein diacetate (DCFH-DA) staining as performed in our previous study [27, 31]. We found that much weak signals of ROS were detected in control oocytes (Fig. 8A). However, heat stress substantially increased the green signals in the cytoplasm of oocytes, which was alleviated by the supplementation of NMN (Fig. 8A). Consistently, the fluorescence intensity of ROS signals in heat-stressed oocytes prominently increased compared to the controls (8.4 ± 0.7, *n* = 38 vs. 31.9 ± 2.6, *n* = 24, *P* < 0.001; Fig. 8B). NMN supplementation effectively reduced ROS levels in heat-stressed oocytes (10.1 ± 0.6, *n* = 55, *P* < 0.001; Fig. 8B).

**Fig. 9:**
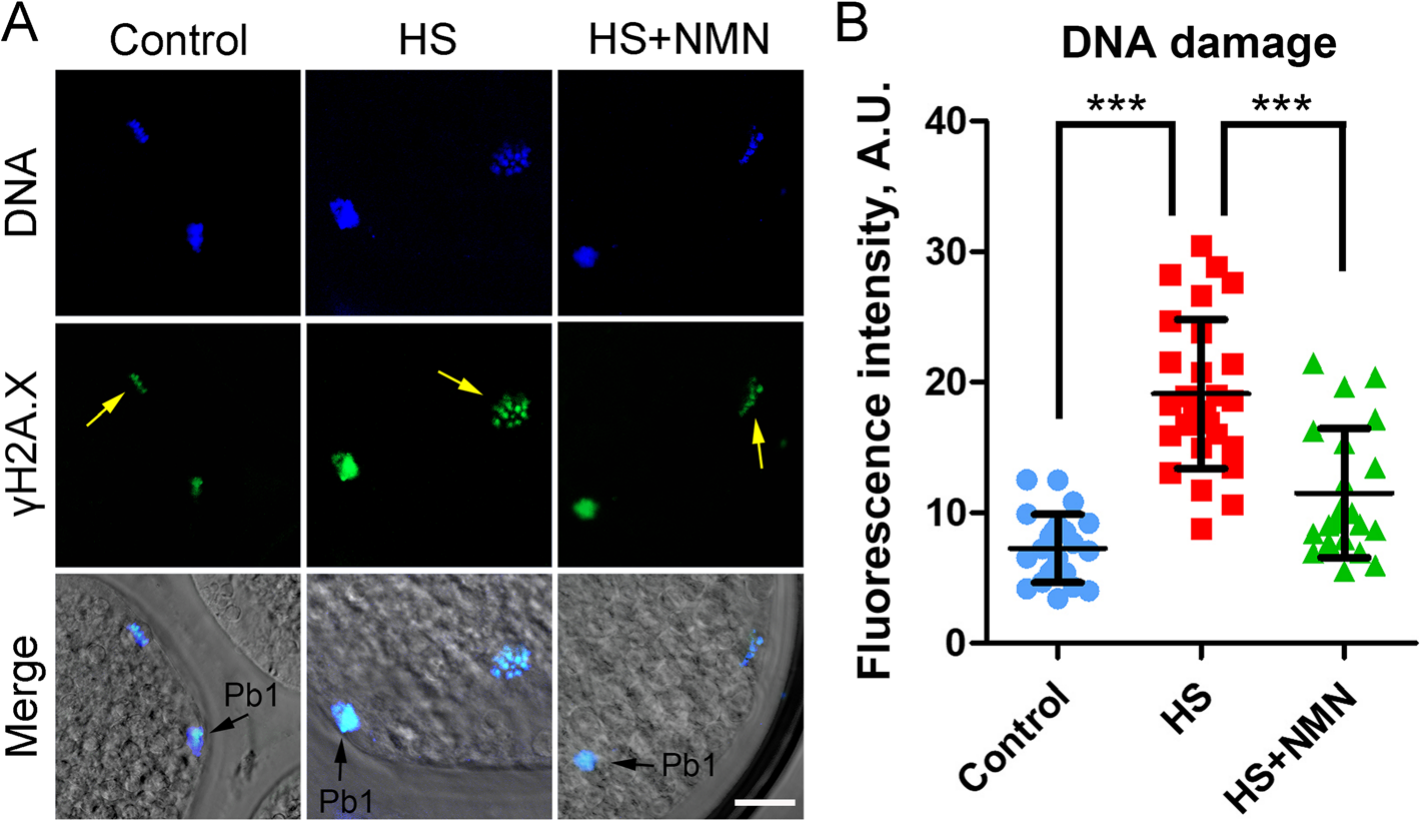
Effects of NMN supplementation on the DNA damage in heat-stressed porcine oocytes. **A** Representative images of accumulated DNA damage in control, heat-stressed and NMN-supplemented oocytes at metaphase II stage. Scale bar, 20 μm. **B** The fluorescence intensity of γH2A.X signals was quantified in control, heat-stressed and NMN-supplemented oocytes. Data were presented as mean value (mean ± SD) of at least three independent experiments. ***P < 0.001

**Fig. 10:**
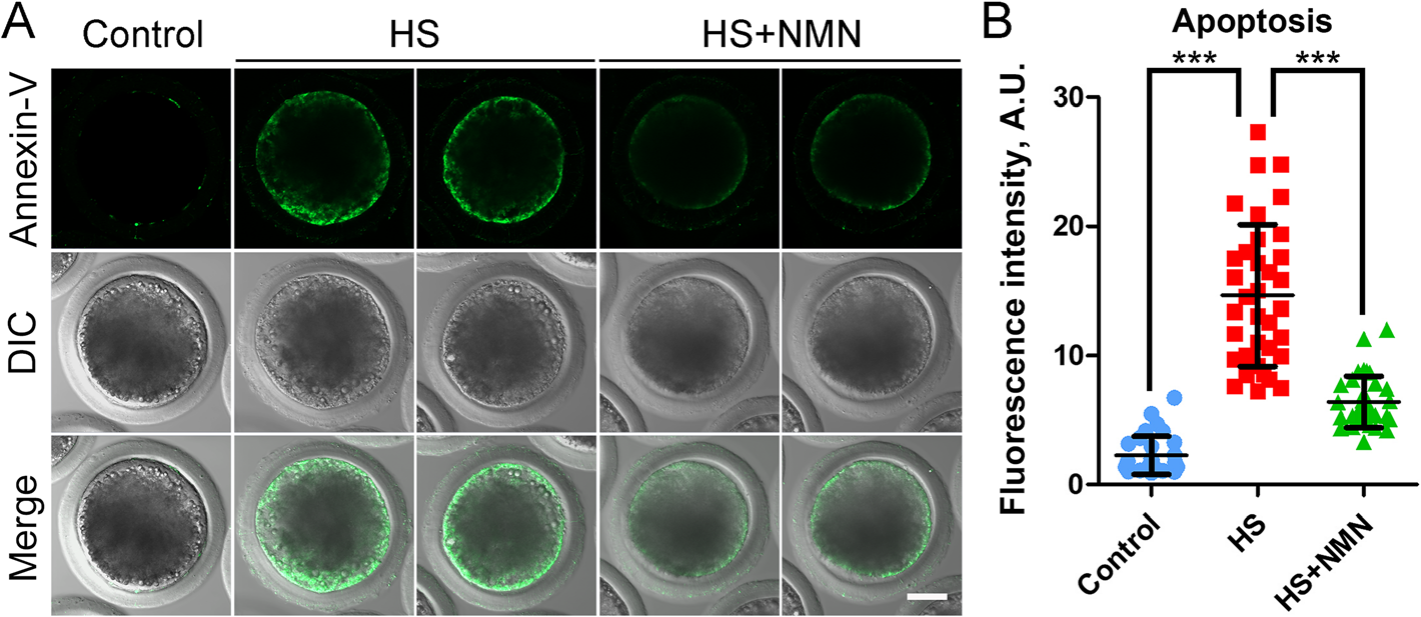
Effects of NMN supplementation on the early apoptosis of heat-stressed porcine oocytes. **A** Representative images of apoptotic oocytes at metaphase II stage in control, heat-stressed and NMN-supplemented groups. Scale bar, 30 μm. **B** The fluorescence intensity of Annexin-V signals was quantified in control, heat-stressed and NMN-supplemented oocytes. Data were presented as mean value (mean ± SD) of at least three independent experiments. ***P < 0.001

Excessive ROS-induced oxidative stress is always related to DNA damage accumulation and early apoptosis in cells. Thus, we stained oocytes with γH2A.X antibody and Annexin-V dye to evaluate the occurrence of DNA damage and apoptosis. We observed that γH2A.X signals in heat-stressed oocytes were significantly higher than those in controls, while NMN supplementation reduced this change (7.3 ± 0.5, *n* = 23, *P* < 0.001 vs. 19.1 ± 1.1, *n* = 28 vs. 12.8 ± 1.3, *n* = 23, *P* < 0.001; Fig. 9A, B). Furthermore, fluorescence imaging and intensity measurement showed that Annexin-V signals were hardly detected in control oocytes, but clearly present on the plasma membrane of heat-stressed oocytes, which were reduced in the NMN-supplemented oocytes (2.3 ± 0.3, *n* = 31, *P* < 0.001 vs. 14.7 ± 0.9, *n* = 35 vs. 6.4 ± 0.3, *n* = 34, *P* < 0.001; Fig. 10A, B). Overall, these results demonstrate that NMN supplementation suppresses the occurrence of apoptosis through reducing levels of ROS and DNA damage in porcine oocytes under heat stress.

### NMN promotes the development of early embryos from the porcine oocytes under heat stress

**Fig. 11:**
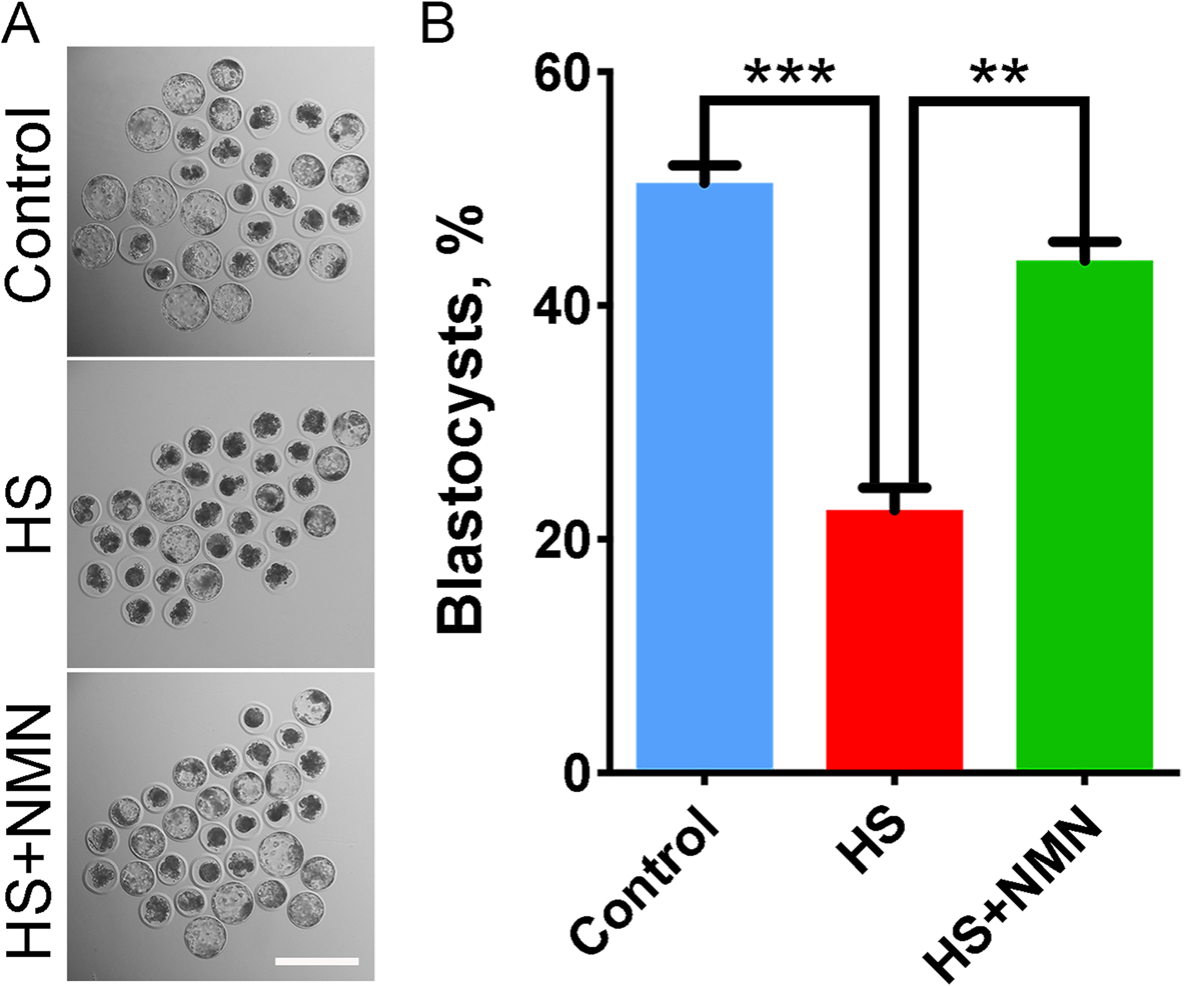
Effects of NMN supplementation on the development of early embryos derived from the heat-stressed porcine oocytes. **A** Representative images of the embryos at blastocyst stage in control, heat-stressed and NMN-supplemented groups. Scale bar, 200 μm. **B** The rate of blastocyst formation was recorded in control, heat-stressed and NMN-supplemented groups. Data were presented as mean value (mean ± SEM) of at least three independent experiments. **P < 0.01, ***P < 0.001

Since NMN improved the quality of heat-stressed porcine oocytes, we further assessed their embryonic development potential by parthenogenetic activation. As shown in Fig. 11, a large number of embryos derived from the control oocytes developed to the blastocysts, while only a few reached this stage in heat-stressed group (50.5 ± 1.5%, *n* = 91 vs. 22.5 ± 1.9%, *n* = 88, *P* < 0.001; Fig. 11A, B). Importantly, NMN supplementation substantially increased the rate of blastocyst formation in heat-stressed group (43.9 ± 1.6%, *n* = 91, *P* < 0.01; Fig. 11A, B), confirming the elevated quality of oocytes under heat stress by NMN.

## Discussion

Higher ambient temperature has various detrimental effects on the organism, such as hyperthermia, endotoxemia and/or systemic inflammation [32, 33]. Increasing studies have shown that the main organelle damaged by heat stress in the cells is mitochondria, and the destruction of mitochondria can lead to cell dysfunction and even necrosis [2]. However, there is no better strategy to solve the damage of heat stress to animals. We previously reported that the restoration of mitochondrial function by NMN supplementation improves the quality of mouse oocytes deteriorated by maternal aging and porcine oocytes exposed to the environmental pollutant ethylene glycol butyl ether [23, 28]. Therefore, we hypothesized that NMN is likely to ameliorate the quality of porcine oocytes under heat stress.

To test this possibility, we firstly assessed the incidence of PBE and cumulus cell expansion, two obvious morphological indicators for the porcine oocyte meiosis. Consistent with previous reports, heat stress impaired the meiotic maturation of porcine oocytes in vitro [34, 35]. Whereas NMN supplementation elevated the rate of PBE and the degree of cumulus cell expansion, validating the beneficial effects of NMN on the oocyte maturation under heat stress. We also evidenced that NMN restored the cytoskeletal structure in heat-stressed porcine oocytes through maintaining the microtubule stability and actin dynamics, which demonstrates that heat stress compromises the porcine oocyte nuclear maturation by disrupting the spindle assembly and actin polymerization, and NMN could recover these meiotic defects.

Another important finding in our study is that NMN promotes the cytoplasmic maturation of porcine oocytes under heat stress. CGs form a uniform layer in the subcortex of oocytes and function in the prevention of polyspermy after fertilization by releasing their component ovastacin out of oocytes to cleave ZP2 at the N-terminus in the zona pellucida [26]. If CGs are induced to exocytose prior to fertilization, ZP2 will be prematurely cleaved to cause the fertilization failure [36]. Thus, the correct distribution of CGs is regarded as a key criterion for the oocyte cytoplasmic maturation. Our data showed that the amount of CGs and their component ovastacin in the subcortical area of porcine oocytes exposed to heat stress were substantially reduced, suggesting that heat stress might weaken the fertilization ability via hindering the cytoplasmic maturation of oocytes. NMN supplementation recovered the dynamics of CGs and ovastacin in porcine oocytes under heat stress. Mitochondrion is a main organelle that provides energy to drive the normal oocyte development [37, 38]. Therefore, mitochondrial distribution is considered as another important indicator for the cytoplasmic maturation of mammalian oocytes. Our findings further documented that NMN restored the oocyte cytoplasmic maturation under heat stress as assessed by the mitochondrial distribution and function.

Lastly, our transcriptome data revealed that spindle organization, cell cycle and DNA damage checkpoint were highly correlated to the NMN-mediated recovery of oocyte maturation under heat stress, which is concordant with our above findings as well as the observation that NMN alleviated the excessive ROS-induced DNA damage accumulation and apoptosis. Consequently, NMN supplementation also promoted the development of embryos derived from the heat-stressed oocytes.

Collectively, we provide several lines of evidence documenting that NMN supplementation is a feasible and effective way to improve the quality of porcine oocytes under heat stress by promoting both nuclear and cytoplasmic maturation.

## Data Availability

The datasets presented in this study can be found in online repositories. The name of the repository and accession number can be found below: https://www.ncbi.nlm.nih.gov/geo/, GSE185338.

## Abbreviations

NMN: Nicotinamide mononucleotide
ROS: Reactive oxygen species
GVBD: Germinal vesicle breakdown
NAD: Nicotinamide adenine dinucleotide
COCs: Cumulus-oocyte complexes
IVM: In vitro maturation
PI: Propidium iodide
DCFH-DA: Dichlorofluorescein diacetate
PBE: Polar body extrusion
CG: Cortical granule
DEGs: Differentially expressed genes
